# Multiple insecticide resistance in *Anopheles gambiae* from Tanzania: a major concern for malaria vector control

**DOI:** 10.1186/s12936-017-2087-2

**Published:** 2017-10-30

**Authors:** William N. Kisinza, Theresia E. Nkya, Bilali Kabula, Hans J. Overgaard, Dennis J. Massue, Zawadi Mageni, George Greer, Naomi Kaspar, Mahdi Mohamed, Richard Reithinger, Sarah Moore, Lena M. Lorenz, Stephen Magesa

**Affiliations:** 1 National Institute for Medical Research (NIMR), Amani Research Center, Muheza, Tanzania; 2 Epidemiology and Public Health Department, Swiss Institute of Tropical and Public Health, Soccinstrase 57, 4002 Basel, Switzerland; 3 University of Basel, Petersplatz 1, 4003 Basel, Switzerland; 4 Ifakara Health Institute (IHI), Bagamoyo, Tanzania; 5 U.S. President’s Malaria Initiative/U.S. Agency for International Development, Dar es Salaam, Tanzania; 6 Department of Mathematical Sciences and Technology, Norwegian University of Life Sciences, Akershus, Norway; 7 Institut de Recherche pour le Développement, Maladies Infectieuses et Vecteurs, Ecologie, Génétique, Evolution et Contrôle, Montpellier, France; 8 RTI International, Dar es Salaam, Tanzania; 9 RTI International, Washington, DC USA; 10 London School of Hygiene & Tropical Medicine, Keppel Street, London, UK

**Keywords:** Insecticide resistance, *Anopheles gambiae*, Malaria, Pirimiphos-methyl, kdr, Tanzania

## Abstract

**Background:**

Malaria vector control in Tanzania is based on use of long-lasting insecticide treated nets (LLINs) and indoor residual spraying (IRS), which both rely on the use of chemical insecticides. The effectiveness of these control tools is endangered by the development of insecticide resistance in the major malaria vectors. This study was carried out to monitor the susceptibility status of major malaria vectors to insecticides used for IRS and LLINs in mainland Tanzania.

**Methods:**

Mosquito larvae were collected in 20 sites of Tanzania mainland in 2015. Phenotypic resistance was determined using standard WHO susceptibility tests. Molecular assay were used to determine distribution of *Anopheles gambiae* sub-species. A microplate assay approach was used for identifying enzyme levels on single mosquitoes from each sites compared with a susceptible reference strain, *An. gambiae* sensu stricto (s.s.) Kisumu strain.

**Results:**

*Anopheles arabiensis* was the dominant malaria specie in the country, accounting for 52% of the sibling species identified, while *An. gambiae* s.s. represented 48%. In Arumeru site, the dominant species was *An. arabiensis*, which was resistant to both pyrethroids (permethrin and deltamethrin), and pirimiphos-methyl, and had significant elevated levels of GSTs, non-specific esterases, and oxidase enzymes. *An. arabiensis* was also a dominant species in Kilombero and Kondoa sites, both were resistant to permethrin and deltamethrin with significant activity levels of oxidase enzymes. Resistance to bendiocarb was recorded in Ngara site where specie composition is evenly distributed between *An. gambiae* s.s. and *An.arabiensis*. Also bendiocarb resistance was recorded in Mbozi site, where *An. gambiae* s.s. is the dominant species.

**Conclusions:**

Overall, this study confirmed resistance to all four insecticide classes in *An. gambiae* sensu lato in selected locations in Tanzania. Results are discussed in relation to resistance mechanisms and the optimization of resistance management strategies.

## Background

Malaria vector control in sub-Saharan Africa is based on the use of long-lasting insecticide-treated nets (LLINs) and indoor residual spraying (IRS), which both rely on the use of chemical insecticides. Malaria transmission and control has been greatly improved by these vector control tools [1]. Since these vector control tools depends on the use of chemical insecticides, for them to remain effective, malaria vectors have to remain susceptible to these insecticides, among other factors. Unfortunately, currently used vector control interventions are dependent on a limited number of insecticides from four chemical classes: organochlorines, organophosphates, carbamates and pyrethroids. Among the four main classes of insecticides used for public health, pyrethroids are the only class of insecticides currently recommended by the World Health Organization (WHO) for use in LLINs [2]. The accomplishments made in malaria control are being threatened by reports of insecticide resistance to all the major classes of insecticides used for public health across sub-Saharan Africa [1]. The two main mechanisms responsible for insecticide resistance are target site insensitivity known as knock down resistance (*kdr*) and metabolic resistance due to elevated levels of insecticide detoxifying enzymes [3].

Unfortunately, despite the ongoing malaria vector control efforts in Tanzania, malaria continues to be a main public health problem with high mortality and morbidity [4]. The major malaria vectors in Tanzania mainland are *Anopheles gambiae* sensu stricto (s.s.) being indoor feeders and *An. gambiae arabiensis* (referred to as *An. arabiensis*) being more outdoor feeders [5]. High LLINs coverage and IRS, has dramatically changed Tanzania vector population whereby the predominant indoor *An. gambiae* s.s. is replaced by *An. arabiensis*, which leads to residual outdoor transmission of malaria [6].

In Government’s efforts to elevate the burden and mortality of malaria, through the National Malaria Control Programme (NMCP) it has in the past decade and a half increased LLINs through universal coverage and increased IRS coverage around the Lake Zone area that has highest malaria prevalence. The distribution of LLINs has targeted the most exposed group (pregnant women and children under 5 years of age), through discounted vouchers issued at antenatal clinics [7, 8], and then by free LLINs delivery campaign in 2010 [8], which was protracted to the general population through a universal coverage LLIN distribution campaign in 2011. In 2013, a school-based LLIN continuous distribution approach (School Net Program) was started in the country’s Southern zone [8] and is still ongoing and expanding to Northern zones. IRS operations were initiated in Kagera region around the Lake Victoria in 2006, and extended to the most of districts around the Lake Zone area in 2011. IRS operations started with using a pyrethroid (lambdacyhalothrin), followed by a carbamate (bendiocarb) in 2009, and an organophosphate pirimiphos-methyl (Actellic 300SC) in 2014.

Previous studies conducted in Tanzania showed wide-spread *An. gambiae* sensu lato (s.l.) resistance to pyrethroids [9–13], and focal resistance to DDT and bendiocarb [9, 10, 12, 14]. The major objective of this study was to continue monitoring the susceptibility status of malaria vectors to insecticides used for IRS (i.e. pirimiphos-methyl, DDT and bendiocarb) and in LLINs (permethrin and deltamethrin). This study also aimed to determine associated resistance mechanisms in sampled mosquito populations. Such information is required when planning future vector control efforts and strategies.

## Methods

### Study design and sites

**Fig. 1:**
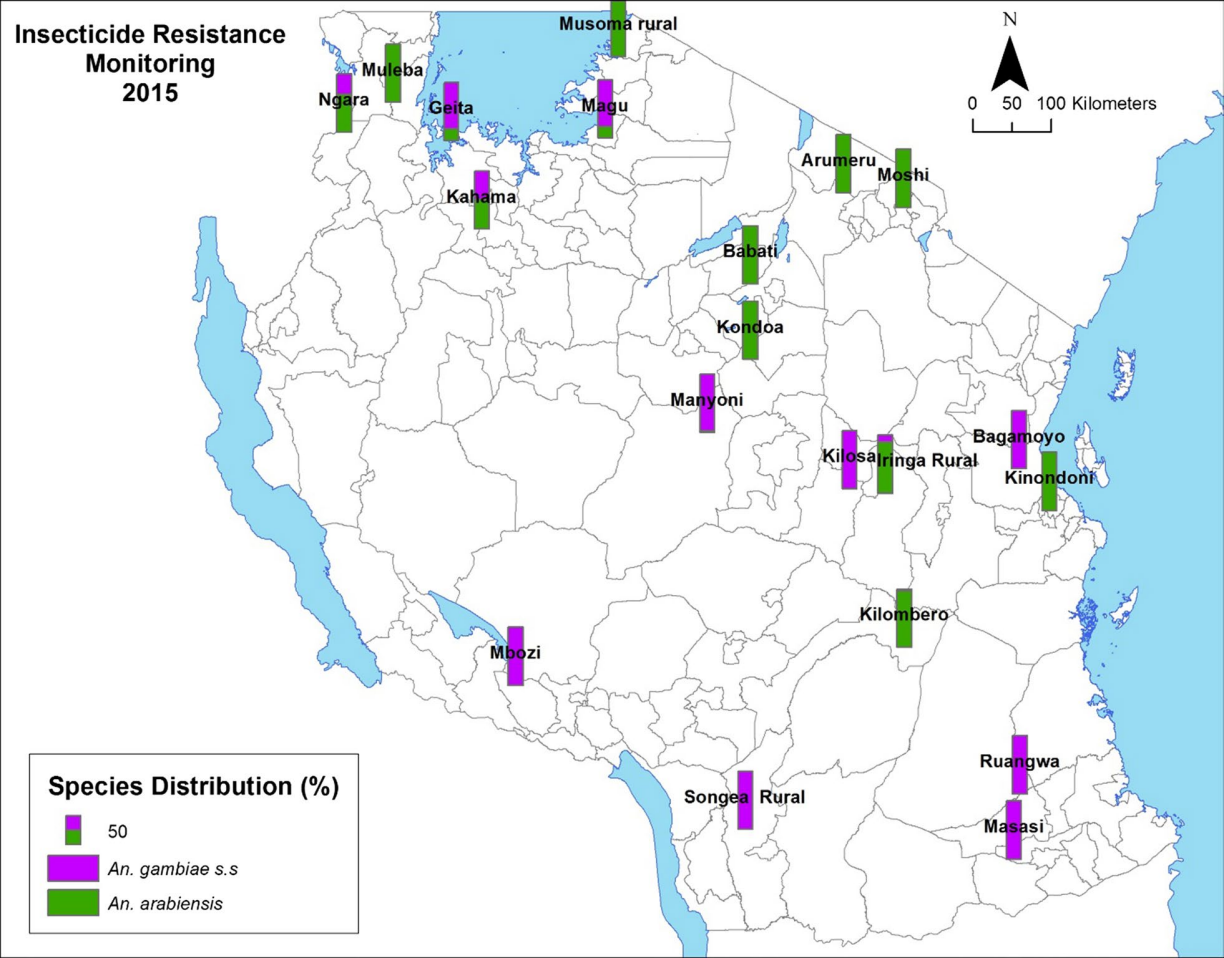
Distribution of a sample of *Anopheles gambiae* s.l. specimens identified to *An. gambiae* s.s. and *An. arabiensis* in surveyed sentinel districts

A cross-sectional countrywide survey was conducted to detect and monitor insecticide resistance in malaria vectors. Field work was carried out between May and June 2015 in 20 established sentinel sites distributed across the country (Fig. 1). These sentinel sites were selected based on WHO criteria and different malaria epidemiological and vector ecological settings, as described previously [15].

### Larval collections and rearing

*Anopheles* larvae were collected from various larval habitats in each study site using a 350 ml dipper. Larvae were transferred into plastic containers (allowing for aeration) and transported in cool boxes to the insectary where they were reared at 27–30 °C and 76 ± 5% relative humidity with a 12 h: 12 h light and dark cycle. The larvae were fed with ground Tetramin® (Tetra, Blacksburg, VA) fish food. The development of the larvae was monitored regularly and pupae were transferred into shallow plastic cups/small beakers using Pasteur pipettes and placed in appropriately labelled cages for adult emergence [16]. Global Positioning System (GPS; Trimble Geoexplorer II, Trimble Navigation Limited, Sunnyvale, CA, USA), was used to record the geographical coordinates of each sampling site.

### WHO insecticide susceptibility tests

Bioassays were carried out using WHO insecticide susceptibility test kits for adult mosquitoes following a standard protocol [16]. Test papers impregnated with the WHO-recommended discriminating dosages of permethrin (0.75%), deltamethrin (0.05%), DDT (4%), pirimiphos-methyl (0.25%) and bendiocarb (0.1%) were used. The quality of the test paper was checked against a laboratory susceptible *An. gambiae* s.s. Kisumu strain at the Amani Medical Research Centre prior to being tested on the wild mosquitoes.

For each susceptibility test, batches of 15–25 adult wild female F1 mosquitoes were transferred into exposure tubes for 1 h. During the 60-min exposure period, the number of mosquitoes knocked down was recorded after 10, 15, 20, 30, 40, 50 and 60 min for pyrethroid and organochlorine insecticides only. At the end of the exposure period mosquitoes were transferred into holding tubes. A cotton pad soaked in 10% sugar was placed on top of the holding tube to avoid death by starvation [16].

Mortality was scored 24 h post-exposure and each test for each sample mosquito population was replicated at least four times. Resistance or susceptibility was defined based on WHO criteria, where 98–100% mortality indicates susceptibility; 90–97% mortality requires further confirmation of possible resistance, and less than 90% mortality indicates resistance [16]. Controls were run with local mosquitoes (one control tube for every test run). When control mortality was scored between 5 and 20%, mean observed mortality was corrected using Abbott’s formula [17]. Tested mosquitoes were preserved with silica gel in 1.5 ml Eppendorf tubes and transported to the Amani Medical Research Centre for further laboratory analysis (i.e. molecular species identification). Adult mosquitoes for biochemical assay were not exposed to insecticides, but were freshly frozen when they were four (4) days old. They were kept under − 80 °C until ready for the assays. The Cryo Express (CX) dry shippers were used to transport these frozen mosquito samples from the field to the laboratory. Storage temperature inside the shipping cavity remained at approximately − 190 °C until the liquid nitrogen evaporates from the absorbent material. From each sentinel district, a minimum of 100, freshly frozen adult female mosquitoes were preserved for subsequent biochemical assays.

### Molecular identification of members of the *Anopheles gambiae* complex

PCR for sibling species were conducted on 100 randomly selected adult mosquitoes from each site. These mosquitoes were individually extracted, according to methods previously described [18]. Individual DNA samples were used for discriminating between *An. gambiae* s.s. and *An. arabiensis* following the PCR-based method described by Scott [19].

### Correlation between resistance levels, species and cross resistance

Correlation between species (*An.arabiensis* and *An. gambiae* s.s.), insecticide resistance levels and pattern of phenotypic cross resistance between insecticides were investigated across all sites. Pearson’s correlation coefficient were calculated for all possible pairwise comparison and then subjected to regression analysis for calculating P value. Correlations showing a r ≥ 1 or ≤ − 1 and P value ≤ 0.05 were considered significant.

### Detection of metabolic resistance mechanisms

Biochemical assays were used to quantify the levels of P450 oxidases, acetylcholinesterase, glutathione S-transferases (GST) and nonspecific esterase (NSE) activity in individual mosquitoes. Individual 4-day old *An. gambiae* s.l. adults, reared under insecticide-free conditions and stored at − 80 °C, were homogenized manually in sodium phosphate buffer (pH 7.2) inside 1.5 ml Eppendorf tubes. For the oxidase assays, preparation of samples and analysis were done according to methods previously described [12]. For P450 assays, preparation of samples and analysis were done according to methods previously described [12]. For the esterase assays preparation of samples and analysis were done according to methods previously described [12]. To measure the level of GST present, 100 μl reduced glutathione was added to 100 μl cDNB. To measure the amount of acetylcholine esterase present, 100 μl mosquito homogenate samples were put in appropriate wells and 100 μl ATCH was added to each well followed by adding 100 μl DTNB to each well.

### Data analysis

Percentage mortality and 95% confidence intervals in WHO susceptibility tests were calculated by the binomial exact method PoLo Plus [20]. Fully susceptible reference strains were also tested in parallel: the Kisumu strain (*An. gambiae* s.s. S form isolated from Western Kenya). For each insecticide, the RR_50_ was calculated as of KDT_50_ of each population divided by the KDT_50_ of the Kisumu strain. The proportions of *An. gambiae* and *An. arabiensis* were determined in each subsample and weighted by the inverse of the sampling fraction (i.e. subsample/total collected) to represent the relative proportion in the total population. For biochemical tests, mean absorbance values of replicate wells for each tested mosquito were converted into enzyme activity and divided by the protein values. The median enzymatic activity was calculated for each test mosquito population and the distribution of enzyme activities was compared between the Kisumu reference strain and the field populations using non-parametric Mann–Whitney tests.

## Results

### Molecular identification of members of the *Anopheles gambiae* species complex

Of 10,340 mosquitoes morphologically identified as *An. gambiae* s.l., 3000 (29%) were subjected to PCR analysis for sibling species identification. Out of 2972 mosquitoes where DNA was successfully amplified, 48 and 52% were identified as *An. gambiae* s.s. and *An. arabiensis* based on the 390 and 315 bp species-specific, diagnostic DNA fragments, respectively. The distribution of the two sibling species at each of the sentinel sites is shown in Fig. 1.

### Resistance spectrum

**Fig. 2:**
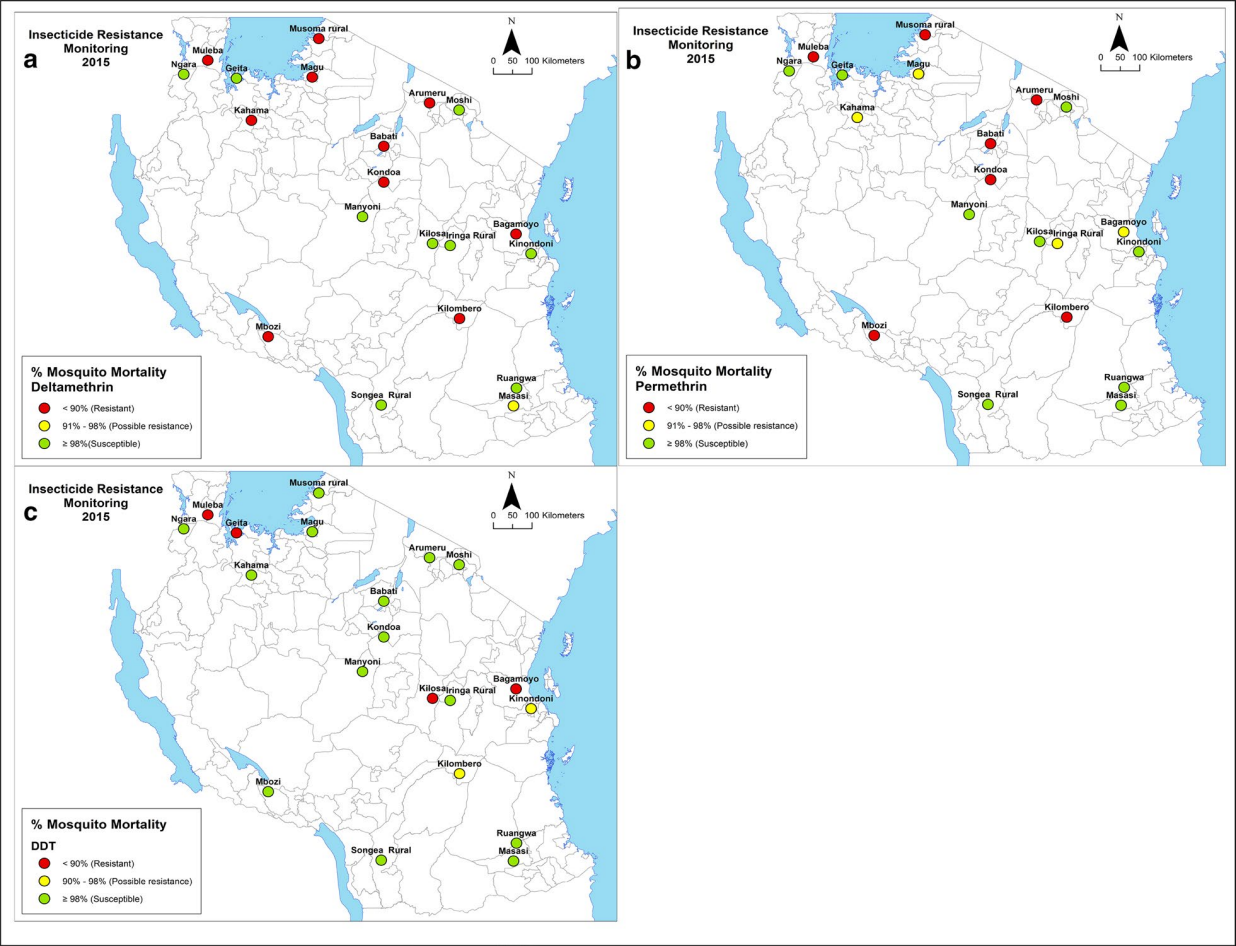
The distribution of **a** deltamethrin **b** permethrin and **c** DDT resistance in 20 sentinel sites across Tanzania

**Fig. 3:**
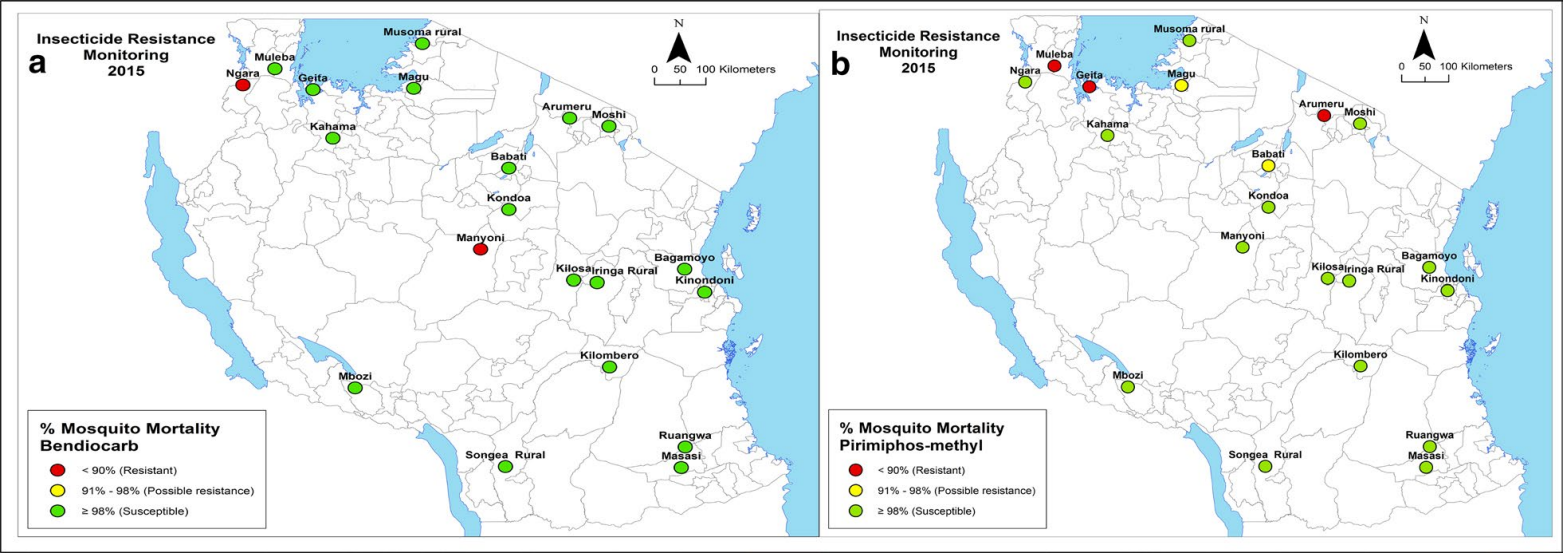
The distribution of **a** bendiocarb and **b** pirimiphos-methyl resistance in 20 sentinel sites across Tanzania

Overall, 10,340 adult mosquitoes reared from larval collections and identified morphologically as *An. gambiae* s.l. were exposed to discriminating insecticide doses. Of the sampled mosquito populations, 5/20 (25%) were found to be resistant to permethrin (range between sites: 19–100%) (Table 1, Fig. 2); 7/20 (35%) to deltamethrin (range between sites: 33.3–100%) (Table 1, Fig. 2); 4/20 (20%) to DDT (range between sites: 59–100%) (Table 1, Fig. 2); 2/20 (10%) to bendiocarb (range between sites: 81–100%) (Table 2, Fig. 3); and 3/20 (15%) to pirimiphos-methyl (range between sites: 82.5–100%) (Table 2, Fig. 3). The median knockdown times (KDT_50_) obtained from time-mortality regression using Probit analysis ranged from 11.6 to 67.0 min for permethrin; 10.2 to 41.9 min for deltamethrin; and 11.6 to 69.5 min for DDT (Table 1).

**Table 1:** Susceptibility status (mortality rates) of *Anopheles gambiae s.l* exposed to the WHO-discriminating concentrations of deltamethrin, permethrin and DDT

Site	Permethrin				Deltamethrin				DDT			
	N	Mortality (%)	SE	KDT_50_ (95% CI)	N	Mortality (%)	SE	KDT_50_ (95% CI)	N	Mortality (%)	SE	KDT_50_ (95% CI)
Arumeru	100	*57*	4	52.3 (45.0–66.0)	100	63	2.1	41.9 (37.1–48.8)	100	*99*	0.1	37.4 (35.4–39.6)
Babati	99	65	2.8	67.0 (57.2–85.3)	100	84	2.2	35.6 (32.2–39.7)	100	100	0	28.2 (21.0–35.8)
Bagamoyo	80	95	1.5	11.9 (11.1–12.7)	80	78	1	14.4 (12.7–15.9)	80	59	1.8	11.6 (10.6–13.4)
Geita	80	98	1	19.6 (18.1–21.2)	60	98.8	1.2	17.2 (15.7–18.7)	80	80	2.4	30.0 (28.5–31.4)
Iringa	100	97	0.3	34.1 (32.3–36.1)	100	98	0.5	32.0 (30.3–33.7)	100	100	0	23.0 (22.1–24.0)
Kahama	60	96.7	1.4	21.7 (18.5–25.1)	60	85	1.9	18.5 (13.9–22.7)	80	100	0	33.9 (30.5–37.2)
Kilombero	100	58	3.2	44.2 (42.4–46.2)	100	66	2.5	40.4 (38.3–42.8)	100	93	1.1	38.1 (36.5–39.7)
Kilosa	80	100	0	28.8 (27.2–30.4)	60	100	0	32.7 (30.6–34.9)	80	71	2.1	50.7 (45.0–60.4)
Kinondoni	100	100	0	54.3 (44.3–75.2)	100	100	0	39.6 (36.5–43.4)	100	91	0.2	69.5 (55.6–99.8)
Kondoa	80	38.8	2.6	50.0 (46.2–55.5)	80	56.8	1.4	36.5 (34.4–38.6)	80	100	0	68.9 (62.3–84.3)
Magu	60	93.3	1.8	28.5 (25.7–31.3)	60	81.7	1	35.8 (32.7–39.5)	60	100	0	41.6 (38.7–44.7)
Manyoni	80	100	0	21.2 (19.6–23.0)	60	93.3	0	22.9 (19.6–26.2)	80	100	0	38.3 (36.0–40.8)
Mbozi	80	100	0	11.6 (10.6–12.4)	80	100	0	13.4 (12.7–14.1)	60	100	0	27.8 (19.2–36.1)
Moshi	100	19	1.9	46.8 (44.5–49.5)	96	33.3	1.3	32.7 (29.7–36.0)	93	100	0	34.2 (32.9–35.6)
Mtwara	80	100	0	11.9 (10.9–12.7)	80	100	0	17.3 (16.5–18.0)	80	100	0	28.9 (27.4–30.7)
Muleba	80	*70*	1.3	44.7 (42.6–47.1)	80	61.3	1.9	36.9 (34.7–39.0)	80	87.5	2.8	35.2 (35.2–36.9)
Musoma–rural	80	60	4.6	51.2 (39.9–90.7)	80	81.3	2.9	38.9 (37.0–40.8)	80	100	0	30.3 (28.8–31.9)
Ngara	60	100	0	16.4 (15.8–16.9)	60	100	0	17.3 (16.0–18.7)	60	100	0	15.8 (15.1–16.5)
Ruangwa	80	100	0	24.6 (22.7–26.5)	80	100	0	20.4 (19.1–21.8)	80	100	0	32.0 (29.2–35.0)
Songea	60	100	0	12.4 (11.2–13.5)	60	100	0	10.2 (9.0–11.2)	80	100	0	26.7 (25.4–28.1)

**Table 2:** Susceptibility status (mortality rates) of *Anopheles gambiae s.l* exposed to the WHO—discriminating concentrations of bendiocarb and pirimiphos-methyl

Site	Bendiocarb			Pirimiphos-methyl		
	N	Mortality (%)	SE	N	Mortality (%)	SE
Arumeru	100	98	0.1	100	87	2.7
Babati	100	100	0	100	94	1.1
Bagamoyo	80	99	0.1	80	100	0
Geita	100	100	0	80	82.5	1.2
Iringa	60	100	0	80	100	0
Kahama	60	100	0	80	98.3	0.1
Kilombero	100	100	0	100	100	0
Kilosa	80	100	0	80	100	0
Kinondoni	100	100	0	100	100	0
Kondoa	80	100	0	80	100	0
Magu	60	100	0	60	95	1
Manyoni	60	100	0	80	100	0
Mbozi	60	81.7	0.3	80	100	0
Moshi	100	98.8	0.2	80	100	0
Mtwara	80	100	0	80	100	0
Muleba	80	98	1.9	80	86.3	1.7
Musoma	80	100	2.9	80	100	0
Ngara	60	81	0	60	100	0
Ruangwa	80	100	0	80	100	0
Songea	80	100	0	60	100	0

### Correlation between resistance levels, species and cross resistance

Significant correlation was found between *An. arabiensis* and pyrethroids (permethrin and deltamethrin); r = 0.66, P value = 0.005. No significant correlation was found between *An. gambiae* s.s. and pyrethroids (r = 0.56, P value = 0.25). As expected, strong correlation was found in the pattern of phenotypic cross resistance between deltamethrin and permethrin (r = 0.82, P value = 0.01).

### Microplate assays of NSE, oxidase and GSTs activity

Biochemical assays were successfully performed on samples from 15 sites comprised of *An. gambiae* s.l. populations. Table 3 shows the means of optical density values for NSEs, mixed function oxidases and GSTs. The results for NSEs show a significantly higher activity in *An. gambiae* s.l. from the Arumeru sentinel site compared to the susceptible Kisumu strain. For mixed function oxidases, the enzyme activity levels were significantly higher in mosquito populations from Bagamoyo compared to the susceptible Kisumu strain. For GSTs, the enzyme activity levels were significantly higher in mosquito populations from Arumeru, Kahama and Kyela.

**Table 3:** Microtitre plate assays optical density values of enzymes activity

Site	Non specific esterases	Oxidases	GSTs
	Mean ± SD	Mean ± SD	Mean ± SD
Kisumu (susceptible strain)	0.95 ± 0.11	0.65 ± 0.19	0.015 ± 0.036
Arumeru	1.58 ± 0.46^a^	0.83 ± 0.12^a^	0.232 ± 0.105^a^
Babati	1.22 ± 0.22	0.63 ± 0.14	0.003 ± 0.001
Bagamoyo	0.84 ± 0.46	0.1 ± 0.43	0.021 ± 0.048
Iringa	0.96 ± 0.52	0.65 ± 0.5	0.001 ± 0.003
Kahama	0.91 ± 0.13	0.6 ± 0.17	0.041 ± 0.116^a^
Kilombero	1.12 ± 0.39	0.96 ± 0.76^a^	0.031 ± 0.034
Kilosa	0.92 ± 0.11	0.51 ± 0.09	0.001 ± 0.002
Kondoa	1.17 ± 0.22	0.91 ± 0.2^a^	0.001 ± 0.008
Kyela	0.88 ± 0.52	0.48 ± 0.31	0.02 ± 0.048^a^
Magu	0.93 ± 0.10	0.64 ± 0.07	0.003 ± 0.001
Musoma	0.99 ± 0.16	0.53 ± 0.26	0.003 ± 0.001
Ngara	0.94 ± 0.17	0.53 ± 0.14	0.003 ± 0.001
Ruangwa	1.10 ± 0.35	0.74 ± 0.75	0.042 ± 0.039
Songea	0.87 ± 0.13	0.81 ± 0.21	0.001 ± 0.002
Muheza	0.95 ± 0.37	0.7 ± 0.15	0.004 ± 0.003

*GST* glutathione S-transferases

^a^Statistically significant levels compared to the susceptible Kisumu strain

## Discussion

The present study aimed at describing the current insecticide resistance status of *An. gambiae* s.l. and the associated resistance mechanisms in Tanzania. The study confirms the previously reported widespread resistance to pyrethroids, as well as focal resistance to DDT and bendiocarb [12, 15, 16, 21, 22]. Additionally, for the first time in Tanzania *An. gambiae* s.l. resistance to pirimiphos-methyl is reported.

The sampled mosquitoes were identified as *An. arabiensis* and *An. gambiae* s.s. in all sites. Replacement of the traditional malaria vector *An. gambiae* s.s. by the more exophagic *An. arabiensis* throughout the country has previously been reported [6, 14, 22]. The national campaign to distribute LLINs and maintaining universal coverage, would only mean dramatic reduction of malaria transmission in these areas. However, malaria remain to have high mortality and morbidity especially to children under 5 years of age and pregnant women [1]. It is important to note that the shift of once used to be the dominant *An. gambiae* s.s. to *An. arabiensis* which is an outdoor feeder, may undermine confidence in LLINs and IRS. Studies have shown that the shift in these sibling species has contributed to the drastic drop in density of *An. gambiae* s.s. relative to *An. arabie*nsis which has led to residual malaria transmission [6]. This poses a potential challenge for vector control, as most available approaches and tools target the endophagic *An. gambiae* s.s. If residual malaria transmission in Tanzania is to be controlled, research on vector control tools targeting the exophagic *An. arabiensis* should be prioritized.

This study showed that pyrethroids resistance is wide spread and observed in 13 sites out of 20 that were surveyed. This concurs with previous reports of the ongoing annual detection and insecticide resistance monitoring programme in Tanzania [15]. The link between pyrethroids resistance and *An. arabiensis*, which now a predominant species in Tanzania, was confirmed by significant correlation between the two variables. *Anopheles arabiensis* showed the highest levels of resistance to all classes of insecticide tested, as demonstrated in Arumeru site. There was no significant correlation between *An. gambiae* s.s. and pyrethroids, this could be due to the limited sample size of the species as they are now being replaced by their sister sibling. The observed resistance to pyrethroids could be attributed to insecticide pressure created by the cumulative effect of insecticide compounds used on insecticide-treated nets [21, 23] livestock pest control and in agriculture [10]. The most common LLINs used in Tanzania are permethrin-impregnated Olyset® (Sumitomo) nets [9]. Additionally, deltamethrin has been used in re-treatment of conventional bednets since the early 2000’s before the introduction of LLINs [9]. These results are in line with previous studies, which reported expansion of pyrethroid resistance in the country [10–14]. The strong correlation found in the pattern of phenotypic resistance between deltamethrin and permethrin across all sites, confirms these two insecticides have the same mode of action, hence resistance impact on the current pyrethroids dependant vector control tools may lead to operational failure. Even though the results reported in this study are of selected sites and from diagnostic tests and do not give the conclusive indication on the impact of insecticide resistance on the current control tools, however they are a first step in identifying the problem which in the future may deem detrimental to malaria control efforts in the country.

Pirimiphos-methyl (Actellic® 300CS, Syngenta) is the only organophosphate used in IRS in Tanzania since 2014. This study reports for the first time in three of 20 sites in Tanzania, *An. gambiae* s.l. resistance to pirimiphos-methyl. The detection of pirimiphos-methyl resistance is of much concern, because this compound is being used as an alternative insecticide after mosquitoes became resistant to pyrethroids and carbamates [10, 13, 14, 21]. Resistance to pirimiphos-methyl was detected in Geita and Muleba, two sites around Lake Victoria, and in Arumeru in the Arusha region. The rapid development of insecticide resistance has been observed in laboratory experiments [24]. Use of the compound of same class as pirimiphos methyl as agrochemicals and for IRS is likely to have contributed to the observed rapid emergence of this insecticide resistance in malaria vectors [25] in the reported sites. Most insecticides used in agriculture are of the same chemical classes, having the same targets and modes of action as those used for vector control [25, 24]. In Tanzania, pirimiphos-methyl is used in several formulations to control agricultural pests in farms and in storage of agricultural produce such as cereals and legumes (Nkya, Unpublished data). Historically, Arumeru district has intensive agriculture with expansive use of insecticides of various classes, but a limited insecticide pressure from vector control activities (Nkya, unpublished data), and hence potentially accounts for the observed high level pirimiphos-methyl resistance; the insecticide pressure created by its use in agriculture [25, 24] might have contributed to resistance developing in malaria vectors from this site. Reports of pirimiphos-methyl resistance in areas that rely on its use for malaria control could have implications on the effectiveness of this malaria vector control intervention. IRS has been implemented in Muleba district since 2006, and during the span of almost a decade, three classes of insecticides have been used for spraying, and now resistance to all three classes of insecticides has been documented [14]. Preliminary 2016 results confirmed pirimiphos-methyl resistance in Arumeru, but pirimiphos-methyl susceptibility was seen in Geita/Muleba, suggesting the resistance detected at those sites in 2015 might not be stable.

In previous studies done in Muleba district, concur with the results of this study. Previously, it has been reported mosquitoes from Muleba to be resistant to bendiocarb, DDT, permethrin and deltamethrin [14], which concurs with the reported resistance to the same insecticides. In terms of species distribution, the previous study in Muleba reported *An. gambiae* s.s. as the predominant species [14], however this study reports *An. arabiensis* as the predominant species. The difference in species distribution between these studies might be due to the once ongoing IRS program in Muleba district, which targeted mostly indoor *An. gambiae* s.s. and not outdoor *An. arabiensis*. Over time this might have contributed to shift in specie composition that is reported in this study. This study did not report the results for *kdr* mutation as they were inconclusive and at a very low frequency. This could be due to fact that the species sampled for this study was predominantly *An. arabiensis* and an absence of *kdr* mutation in this species had been reported in previous studies [11].

The underlying resistance mechanisms reported in this study is predominantly metabolic resistance which is the mechanism mostly associated with *An. arabiensis* [11, 14]. Elevated levels of P_450_ oxidases, NSEs and GSTs have been reported to be associated with insecticide resistance across all classes of insecticides [10, 25–28]. In all of the analysed mosquitoes from all sites, only four sites had significantly elevated levels of GSTs, P_450_ oxidase and NSEs as compared to the susceptible Kisumu strain. NSEs and GSTs, were detected in significant levels in mosquito populations from Arumeru. Elevation of NSEs and GSTs have been previously linked to organophosphate resistance [10, 25–30], thus corroborating the findings of this study. In the three sites where vectors demonstrated pirimiphos-methyl resistance, they were also found to have elevated levels of GSTs, NSEs, acetylcholinesterase and mixed function oxidases. Moreover, elevated levels of these enzymes were also reported from other sites that did not have pirimiphos-methyl resistance, but showed resistance to pyrethroids, DDT and bendiocarb. The general assumption is that insecticide resistance give selection pressure to all insecticides with similar mode of action ineffective. This is not true when it comes to metabolic resistance mechanisms, as some P450 s enzymes show specificity for type I pyrethroids (such as permethrin) or typeII pyrethroids (such as deltamethrin) [31, 32]. This further confirms that metabolic resistance may indeed be associated with resistance to different classes of insecticides. As the various insecticide classes used for malaria vector control have been frequently used for crop protection, this may explain why resistance to most insecticides develops so rapidly in malaria vectors in Tanzania.

## Conclusion

Resistance to all four classes of insecticide that are used for malaria vector control purposes has been identified in populations of *An.s gambiae* s.l. from selected locations in Tanzania. As no new insecticides classes are likely to be approved for vector control in the near future, increasing effective resistance management plans is critical to continue to control malaria in Tanzania and globally. This study provides an update on the expanse of resistance and associated mechanisms in the main malaria vectors found in Tanzania. Findings of this study, clearly show the presence of metabolic resistance mechanisms in malaria vectors and cross resistance for insecticide. The widespread pyrethroid resistance and reports of pirimiphos methyl resistance, co-occur in the same *An. gambiae* s.l. populations and may impact LLINs and IRS efficacy, the two main approaches for malaria control in Tanzania. The occurrence of pirimiphos-methyl resistance in malaria vectors is alarming, further resistance monitoring is needed to determine whether the presence of pirimiphos-methyl resistance is a transient finding or an established phenomenon. Also to continue investigating and understand the selection pressures and mechanism underlying the observed phenotypic resistance to pirimiphos-methyl. Further understanding the potential epidemiological impact of insecticide resistance in malaria control, including through studies linking insecticides resistance to control failure, will be critical. Continued monitoring of the selection and spread of insecticide resistance and its underlying mechanisms will help reducing the likelihood of potential vector control failures and improving resistance management strategies.

## Abbreviations

LLIN: long-lasting insecticide-treated net
IRS: indoor residual spraying
DDT: 4,4′-(2,2,2trichloroethane-1,1-diyl)bis(chlorobenzene)
VGSC: voltage-gated sodium channel
WHO: World Health Organization
KDT: knock down time
KD: knock down
NSE: non-specific esterases
GSTs: glutathione S-transferases
Ace-1: acetylcholinesterase
kdr: knock down resistance mutation
gDNA: genomic DNA
cRNA: complementary amplified RNA
P450: cytochrome P450 monooxygenase enzymes
